# Generation and Characterization of a New FRET-Based Ca^2+^ Sensor Targeted to the Nucleus

**DOI:** 10.3390/ijms22189945

**Published:** 2021-09-14

**Authors:** Luisa Galla, Nicola Vajente, Diana Pendin, Paola Pizzo, Tullio Pozzan, Elisa Greotti

**Affiliations:** 1 Neuroscience Institute, National Research Council (CNR), 35131 Padua, Italy; luisa.galla@unipd.it (L.G.); nicola.vajente@studenti.unipd.it (N.V.); diana.pendin@unipd.it (D.P.); paola.pizzo@unipd.it (P.P.); tullio.pozzan@unipd.it (T.P.); 2 Department of Biomedical Sciences, University of Padua, 35131 Padua, Italy; 3 Venetian Institute of Molecular Medicine (VIMM), 35131 Padua, Italy

**Keywords:** calcium, nucleus, nuclear, FRET-based probe, endoplasmic reticulum, Cameleon, IP_3_ receptor, SOCE

## Abstract

Calcium (Ca^2+^) exerts a pivotal role in controlling both physiological and detrimental cellular processes. This versatility is due to the existence of a cell-specific molecular Ca^2+^ toolkit and its fine subcellular compartmentalization. Study of the role of Ca^2+^ in cellular physiopathology greatly benefits from tools capable of quantitatively measuring its dynamic concentration ([Ca^2+^]) simultaneously within organelles and in the cytosol to correlate localized and global [Ca^2+^] changes. To this aim, as nucleoplasm Ca^2+^ changes mirror those of the cytosol, we generated a novel nuclear-targeted version of a Föster resonance energy transfer (FRET)-based Ca^2+^ probe. In particular, we modified the previously described nuclear Ca^2+^ sensor, H2BD3cpv, by substituting the donor ECFP with mCerulean3, a brighter and more photostable fluorescent protein. The thorough characterization of this sensor in HeLa cells demonstrated that it significantly improved the brightness and photostability compared to the original probe, thus obtaining a probe suitable for more accurate quantitative Ca^2+^ measurements. The affinity for Ca^2+^ was determined in situ. Finally, we successfully applied the new probe to confirm that cytoplasmic and nucleoplasmic Ca^2+^ levels were similar in both resting conditions and upon cell stimulation. Examples of simultaneous monitoring of Ca^2+^ signal dynamics in different subcellular compartments in the very same cells are also presented.

## 1. Introduction

The pleiotropic effects of Ca^2+^ changes on cell functions depend on their amplitude, duration, and subcellular localization as well as on their origin, i.e., whether they are caused by the influx of Ca^2+^ across the plasma membrane (PM) or its release from intracellular stores [1]. The high interconnection between global and localized Ca^2+^ dynamics is intrinsic in this signaling pathway, in which several organelles participate in the activation, modulation, and functional effects.

The central role of nuclear Ca^2+^ dynamics in the regulation of key cellular functions is widely recognized. For example, the amplitude and duration of Ca^2+^ increase in the nucleoplasm are responsible for differential gene expression regulation, triggering the activation or inhibition of different cellular pathways [2].

The nuclear content is isolated from the cytoplasm thanks to the presence of a double membrane, namely the inner and outer nuclear membrane (INM and ONM, respectively) called nuclear envelope (NE), which is in continuity with the ER membrane. The NE is impermeable to molecules larger than 50–60 kDa, whose entrance into the nucleus depends on specific targeting sequences (nuclear localization signals, NLS) and by the presence of nuclear pore complexes (NPCs). On the contrary, ions and other small molecules, including Ca^2+^, should freely move from the cytosol to the nucleus and vice versa [3].

Although some recent studies suggest the existence of a specific molecular Ca^2+^ toolkit at the INM and ONM that contributes to generating a cytosol-independent Ca^2+^ signal in the nucleus (reviewed in [4]), important technical issues plague these conclusions. For example, the continuity between ER membrane and NE makes it difficult to discern a possible differential localization of Ca^2+^ handling molecules in these two subcompartments. In addition, and most relevantly, NPCs allow the rapid entrance of commonly used cytosolic dyes into the nucleus, but the biophysical properties of most fluorescent Ca^2+^ probes, in particular the Ca^2+^ affinity (K_d_), are modified by the nuclear environment [5], resulting in non-Ca^2+^-dependent fluorescence differences between the nucleus and cytosol. Finally, chemical dyes can be in part trapped in the ER lumen and thus within the NE, and the high [Ca^2+^] in this compartment can be misinterpreted as a localized high nucleoplasmic [Ca^2+^] [6].

For these reasons, nucleus-targeted genetically encoded Ca^2+^ indicators (GECIs) appear the best choice to investigate nuclear Ca^2+^ homeostasis as they ensure a very specific localization. Their molecular weight (usually above 50 kDa) and, for some probes, the binding to nuclear structures prevent their diffusion into the cytosol through NPCs. Different GECIs have been developed in recent years [7,8,9,10,11,12,13,14,15]. However, these probes have not been thoroughly characterized in terms of photochemical characteristics and, most importantly, in situ Ca^2+^ affinity.

Here, we generated a novel nuclear-targeted Ca^2+^ probe based on the previously generated H2BD3cpv [13]. H2BD3cpv is a FRET-based sensor that is localized in the nucleus thanks to the fusion between part of histone 2B and the cytosolic Cameleon D3cpv. This GECI is ratiometric, has a single and suitable K_d_ for Ca^2+^ in vitro, and offers a good dynamic range. To maximize the probe performance, we corrected some drawbacks of H2BD3cpv, i.e., the low fluorescence of donor ECFP and its photobleaching, by substituting the ECFP with mCerulean3 and by modifying the D3 domain [16]. We thoroughly characterized this new nuclear-targeted Cameleon in situ in HeLa cells, demonstrating that the probe based on mCerluean3 significantly improved the brightness and photostability. Moreover, we measured its K_d_ for Ca^2+^ in situ and observed the presence of a second, very low K_d_. Finally, the new probe, together with other sensors targeted to mitochondria and ER, was successfully used to simultaneously monitor nuclear and organelle Ca^2+^ signaling in response to various cell stimuli.

## 2. Results

### 2.1. Generation of H2BD3mCerulean3+16

We have previously generated and used nucleus-targeted Cameleons, such as H2BD1cpv [8] and H2BD3cpv [13]. Typically, D1-based Cameleons are characterized by a double affinity for Ca^2+^ that complicates quantitative Ca^2+^ measurements, whereas D3-based Cameleons have a single K_d_ [17] but are plagued by other problems (e.g., low brightness). In order to improve the photophysical properties of the H2BD3cpv probe, we adopted a strategy that has been successfully applied in the past to ameliorate the mitochondria-targeted version of D3cpv [16]. Starting from H2BD3cpv, the donor was substituted and the linker between the two Ca^2+^-responsive elements, Calmodulin and M13, was elongated with 16 glycine residues to generate H2BD3mCerulean3+16. Indeed, this approach has been successfully employed in the recovery of the dynamic range reduction induced by the donor substitution of the mitochondrial Cameleon 4mtD3mCerulean3+16 that we previously generated [16].

**Figure 1:**
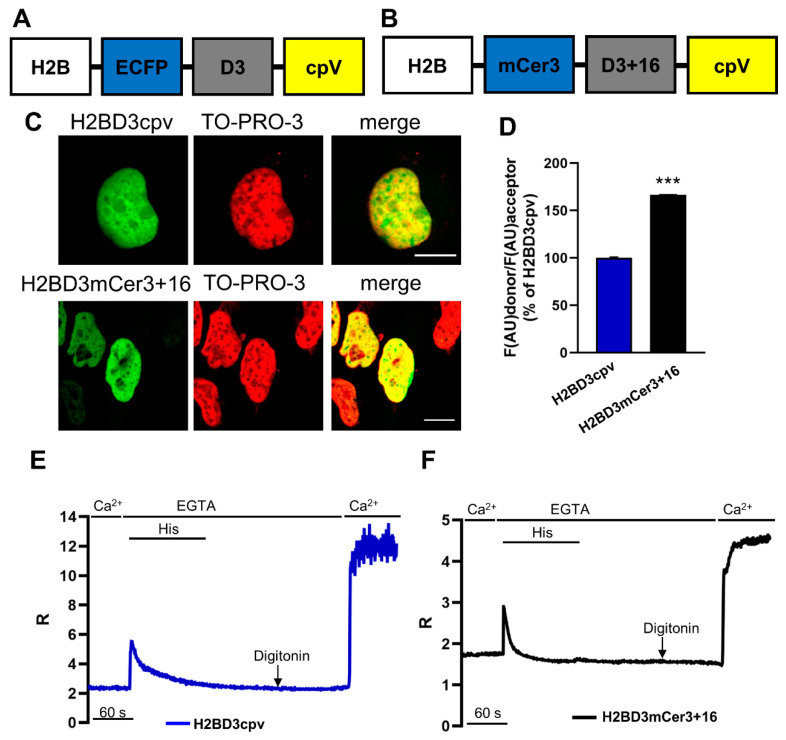
Generation and characterization of a new nuclear Ca^2+^ probe based on D3cpv. (**A**,**B**) Schematic representation of the nucleus-targeted D3cpv-based constructs H2BD3cpv (**A**) and H2BD3mCerulean3+16 (**B**). (**C**) H2BD3cpv (top) and H2BD3mCerulean3+16 (bottom) correctly localize in the nucleus. Confocal images of HeLa cells expressing H2BD3cpv or H2BD3mCerulean3+16 probe (green) and labelled with TO-PRO-3 dye (red). The yellow color represents the superimposition of the Cameleon and TO-PRO-3 signals. Scale bar, 10 µm. (**D**) The bar chart represents the mean ± SEM of ECFP and mCerulean3 fluorescence, normalized to cpV fluorescence, in HeLa cells expressing nuclear Cameleon probes. *n* ≥ 69 cells for each condition. Data are shown as % of H2BD3cpv. (**E**,**F**) Representative kinetics of nuclear R values in a single HeLa cell expressing H2BD3cpv (**E**, blue) or H2BD3mCerulean3+16 (**F**, black) stimulated with histamine (His, 100 µM) in a Ca^2+^-free extracellular medium, then permeabilized with digitonin (100 µM) in an intracellular medium containing EGTA (600 µM), and finally perfused with an intracellular medium containing CaCl_2_ (3 mM). Data are plotted as R, as defined in Materials and Methods. *** *p* < 0.001.

The scheme of H2BD3cpv and of H2BD3mCerulean3+16 constructs is shown in Figure 1A,B. The nuclear localization was verified by confocal microscopy, and the typical pattern of fluorescence obtained in cells transiently expressing H2BD3cpv and H2BD3mCerulean3+16 is presented in Figure 1C. In both cases, the nuclear localization is demonstrated by the overlap of sensor fluorescence with the staining of the nucleus obtained using the nucleic acid marker TO-PRO-3 (Figure 1C).

To check whether the brightness improvement of mCerulean3 (compared to ECFP [18]) was retained in the new probe, HeLa cells were transfected with H2BD3cpv or H2BD3mCerulean3+16, and the fluorescence intensity at 480 nm (the peak emission wavelength of the donor) was measured. Because the fluorescence intensity also depends on the expression levels of the fluorescent sensor, we exploited the fluorescence intensity of cpV emission (excited at 512 nm, emission 535 nm), present in both probes, as normalizing factor for differential expression of donor protein. The mean fluorescence intensity in cells expressing H2BD3mCerulean3+16 was 66% higher than that of cells expressing H2BD3cpv (Figure 1D), confirming the greater brightness of the donor in H2BD3mCerulean3+16 compared to that in H2BD3cpv.

The functionality of both probes was then compared in living HeLa cells challenged with the inositol trisphosphate (IP_3_)-generating agonist histamine (His, Figure 1E,F). At the end of the experiment, probe calibration was carried out. HeLa cells were permeabilized with digitonin and then perfused with an intracellular medium, in the absence of Ca^2+^ and containing 600 µM ethylene glycol tetra acetic acid (EGTA), to obtain the minimum ratio (R) value (R_min_). Notably, despite using a concentration of digitonin that is sufficient to release most of the soluble cytoplasmic proteins into the medium, nearly all the fluorescence of H2BD3mCerulean3+16 and H2BD3cpv remained trapped in the nucleus. Finally, to achieve the maximum R value (R_max_), 3 mM CaCl_2_ was added to the medium.

Qualitatively, the behavior of the two probes was similar, with two important differences: (i) the absolute R values were different with the two probes (Figure 1E,F); (ii) H2BD3cpv showed a high noise of the trace at R_max_ when the ECFP signal was at its minimum (Figure 1E,F).

### 2.2. Biophysical Characterization of Nuclear Cameleons

**Figure 2:**
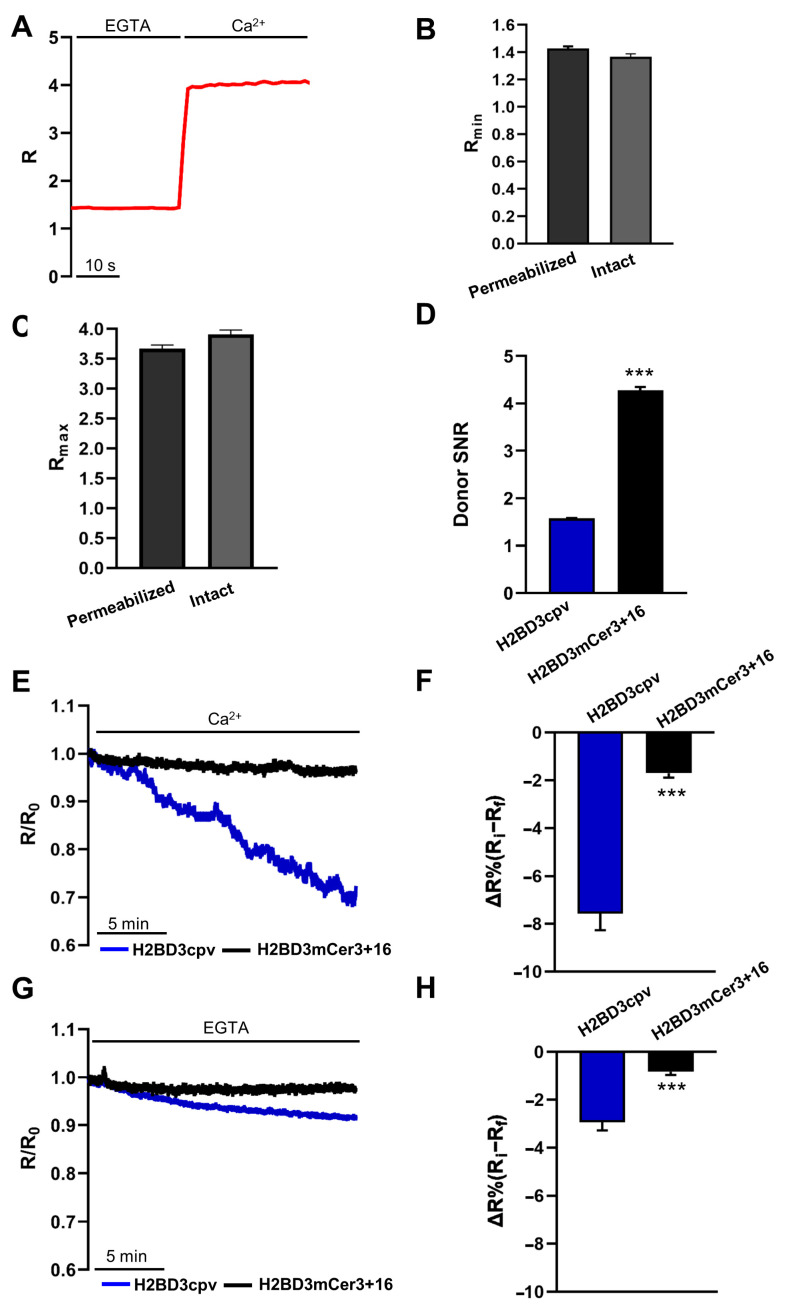
Biophysical characterization of nuclear Cameleons. (**A**) HeLa cells expressing H2BD3mCerulean3+16 were permeabilized with digitonin (100 μM) for 1 min in an intracellular medium containing EGTA (600 μM). Cells were then perfused with EGTA (600 μM) to record R_min_ values and then with CaCl_2_ (3 mM) to obtain R_max_ values. Data are plotted as R/R_0_, as described in Materials and Methods. (**B**) The bar chart shows the R_min_ values as mean ± SEM of *n* ≥ 38 cells calculated in permeabilized (dark gray) or intact (light gray) HeLa cells expressing H2BD3mCerulean3+16. (**C**) The bar chart shows the R_max_ values as mean ± SEM of *n* ≥ 31 cells calculated in permeabilized (dark gray) or intact (light gray) HeLa cells expressing H2BD3mCerulean3+16. (**D**) The bar chart shows the donor SNR of the two nuclear probes as mean ± SEM of *n* ≥ 53 cells for each condition. (**E**,**G**) The representative traces show R changes over time, normalized to the initial R (R_i_), measured by illuminating the sample every second for 20 min in HeLa cells expressing H2BD3cpv (blue) or H2BD3mCerulean3+16 (black) in basal (**E**) or minimal (**G**) FRET condition. (**F**,**H**) The bar charts show the mean ± SEM of ΔR% of the two probes, where ΔR% is calculated as % of the reduction in R between its initial value (R_i_) and its value after 20 min (R_f_), obtained under basal FRET condition (*n* ≥ 30 cells) (**E**) or minimal FRET condition (*n* ≥ 29 cells) (**G**). *** *p* < 0.001.

A key parameter considered for the use of ratiometric GECIs is their dynamic range. In FRET-based GECIs, the dynamic range is calculated as R_max_/R_min_, where R_max_ is the R obtained under Ca^2+^ saturating conditions, while R_min_ is the R obtained in the absence of Ca^2+^ and in the presence of EGTA. To evaluate this parameter in situ, both intact and pre-permeabilized HeLa cells expressing the nuclear probes were used. Pre-permeabilized cells were perfused with intracellular medium containing EGTA to obtain R_min_ and then with the medium containing 3 mM CaCl_2_ to obtain R_max_ (Figure 2A). In intact cells, R_min_ was obtained by loading cells with BAPTA-AM in the absence of extracellular Ca^2+^ and in the presence of EGTA, whereas R_max_ was obtained by treating intact cells with 10 µM ionomycin and 5 mM CaCl_2_ in the medium. For H2BD3mCerulean3+16, the value of R_min_ (Figure 2B) and R_max_ (Figure 2C) as measured in permeabilized cells was not significantly different from that of intact cells.

The dynamic range of H2BD3mCerulean3+16 (R_max_/R_min_ = 2.95 ± 0.06, Table 1) was lower than that of H2BD3cpv (R_max_/R_min_ = 4.94 ± 0.09), as previously observed for the same probes expressed in the cytoplasm and in the mitochondrial matrix. However, the estimation of H2BD3cpv dynamic range is largely artificial and depends on the very low fluorescence intensity of the H2BD3cpv (hardly distinguishable from the background) at high [Ca^2+^]. Indeed, whenever the signal-to-noise ratio (SNR) of the two probes at high [Ca^2+^] was directly assessed (Figure 2D) as ratio between the ECFP fluorescence signal (at 480 nm) and the background, the improvement provided by the substitution of ECFP with mCerulean3 was clear.

Another parameter that we previously found improved upon donor substitution was the probe photostability [16]. To evaluate a possible amelioration, HeLa cells expressing the ECFP-based or mCerulean3-based nuclear Cameleon were imaged for 20 min under identical conditions of illumination and image acquisition. We recorded changes in R in permeabilized cells in basal [Ca^2+^] (basal FRET) in intact cells (see Materials and Methods) or in the absence of external Ca^2+^ (R_min_, minimal FRET). In accordance with our previous observations [16], we found a faster decrease of R in cells expressing H2BD3cpv compared to that observed in cells expressing H2BD3mCerulean3+16, both under basal (Figure 2E,F) and minimal (Figure 2G,H) FRET.

### 2.3. In Situ Titration of H2BD3mCerulean3+16

**Figure 3:**
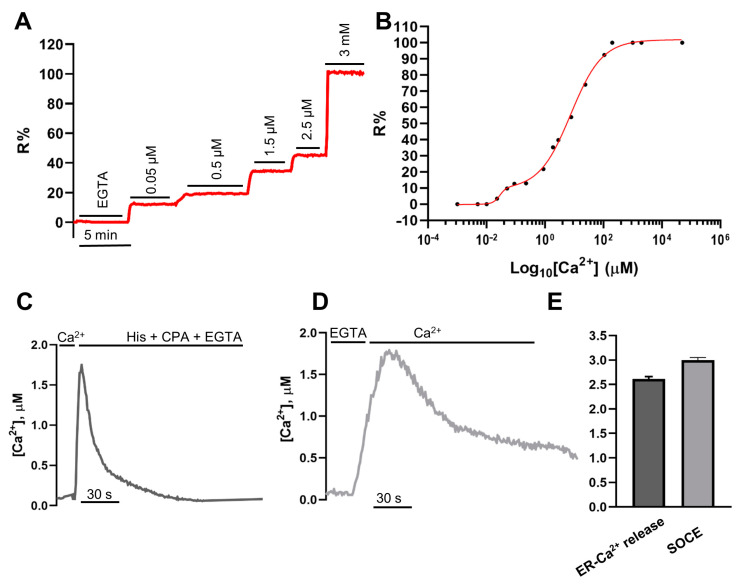
H2BD3mCerulean3+16 Ca^2+^ affinity estimation (**A**,**B**). (**A**) Representative kinetic of R% in permeabilized HeLa cells transiently expressing the H2BD3mCerulean3+16 nuclear probe. Where indicated, digitonin-permeabilized cells were perfused with an intracellular medium without energy sources and containing different [Ca^2+^]. (**A**) In the representative trace, 0.05, 0.5, 1.5, and 2.5 µM CaCl_2_ were used; 3 mM CaCl_2_ was added at the end to reach maximal FRET values. (**B**) In situ Ca^2+^ titration of H2BD3mCerulean3+16 along with the corresponding fit of the data plotted as mean ± SEM (*n* ≥ 7) cells for each [Ca^2+^]. Nuclear [Ca^2+^] upon different cell stimuli (**C**–**E**). Representative kinetics of nuclear [Ca^2+^] changes upon ER Ca^2+^ release (**C**) or SOCE activation (**D**) in HeLa cells expressing H2BD3mCerulean3+16. Data are presented as [Ca^2+^], mean ± SEM. (**E**) The bar chart shows nuclear Ca^2+^ peaks elicited by histamine and cyclopiazonic acid (CPA) application (dark gray) or SOCE activation (light gray) in HeLa cells as mean ± SEM of *n* = 43 cells.

**Figure 4:**
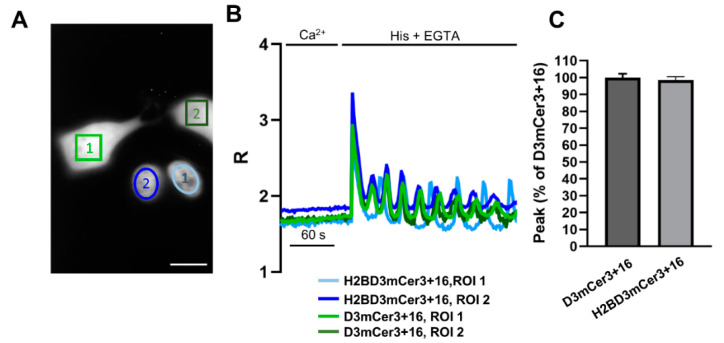
Simultaneous imaging of cytosolic and nuclear Ca^2+^ kinetics. (**A**) Representative image of the acceptor channel of cocultured HeLa cells transfected with H2BD3mCerulean3+16 or D3mCerulean3+16. (**B**) Representative kinetics of nuclear and cytosolic R values, measured by recording the signal from the indicated regions of interest (ROIs), in HeLa cells expressing either H2BD3mCerulean3+16 (light blue circle 1, dark blue circle 2) or D3mCerulean3+16 (light green rectangular 1, dark green rectangular 2) and stimulated with histamine (His, 100 μM), as described in Figure 1C. (**C**) The bar chart shows the mean cytosolic (dark gray) and nuclear (light gray) Ca^2+^ peak values elicited by histamine application in HeLa cells expressing the D3mCerulean3+16 (dark gray) or H2BD3mCerulean3+16 (light gray) as mean ± SEM of *n* ≥ 17 cells.

In situ determination of K_d_ for Ca^2+^ of H2BD3mCerulean3+16 was then carried out in HeLa cells. This parameter is essential for quantitative Ca^2+^ measurements, allowing the conversion of R values into absolute [Ca^2+^]. To evaluate the K_d_, HeLa cells expressing H2BD3mCerulean3+16 were permeabilized with digitonin (100 µM) in an intracellular medium in the absence of external Ca^2+^ and energy sources and containing 600 µM EGTA. The cells were then exposed to different Ca^2+^ concentrations ranging from 20 nM to 3 mM (Figure 3A). By plotting R% values against log_10_[Ca^2+^], we found a double affinity for Ca^2+^ [19], and the calculated apparent K_d_s for H2BD3mCerulean3+16 were 0.03 ± 0.01 μM and 7.57 ± 0.81 μM (Figure 3B and Table 1). The two affinities for Ca^2+^ were unexpected and differed from that of the same probe expressed in the cytoplasm (D3mCerulean3+16) [16]. The calculated K_d_s were then exploited to estimate the [Ca^2+^] of the peaks reached in the nucleus upon maximal release of Ca^2+^ from the ER induced by IP_3_R (IP_3_ receptor) stimulation and SERCA (sarco/endoplasmic reticulum ATPase) blockade (Figure 3C,E) or upon store-operated Ca^2+^ entry (SOCE) activation (Figure 3D,E) [20]. In these HeLa cells, the mean [Ca^2+^] value of the nuclear Ca^2+^ peak elicited by either ER Ca^2+^ release or SOCE activation (Figure 3E) was 2.61 ± 0.3 µM and 2.99 ± 0.3 µM, respectively, similar to that observed with cytosolic D3mCerulean3+16 probe (see Figure 4B).

### 2.4. Simultaneous Monitoring of Nuclear and Cytosolic Ca^2+^ Signaling

To corroborate the notion that the monitoring of nuclear [Ca^2+^] can be exploited as a mirror of cytosolic [Ca^2+^], we cocultured HeLa cells transfected separately with H2BD3mCerulean3+16 or cytosolic D3mCerulean3+16 (Figure 4A). We then selected a field with cells expressing both probes (Figure 4A) and stimulated Ca^2+^ increase by histamine addition (Figure 4A–C). As shown in Figure 4, both the kinetics (Figure 4B) and the amplitude (Figure 4B,C) of cytosolic and nuclear [Ca^2+^] were very similar even though they were measured in cells expressing different probes. In particular, the first peaks (nuclear or cytosolic) were practically indistinguishable, while the following oscillations were asynchronous (Figure 4B) as expected because they came from different cells.

### 2.5. Simultaneous Monitoring of Nuclear and Organelle Ca^2+^ Signaling in the Same Cell

**Figure 5:**
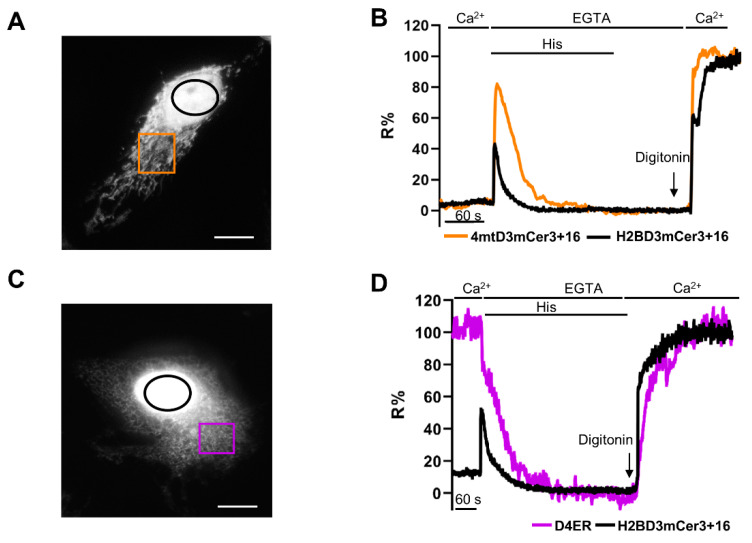
Simultaneous imaging of nuclear and organelle Ca^2+^ kinetics. (**A**–**C**) Representative image of the acceptor channels of HeLa cells coexpressing H2BD3mCerulean3+16 (black) and 4mtD3mCerulean3+16 (**A**, orange) or D4ER (**C**, purple). The fluorescence of H2BD3mCer3+16 was saturated to better visualize mitochondria (**A**) and ER (**C**) fluorescence signals. (**B**–**D**) Representative kinetics of nuclear and mitochondria (**B**) or ER (**D**) R% values in a HeLa cell coexpressing H2BD3mCerulean3+16 and 4mtD3mCerulean3+16 (**B**) or D4ER (**D**) stimulated with histamine, as described in Figure 4B. Data are presented as R% values, as described in Section 4.

We then tested the suitability of the new probe for the simultaneous measurement of nuclear and organelle [Ca^2+^] changes in the very same cell. We first induced ER Ca^2+^ release in HeLa cells coexpressing the mitochondrial matrix-targeted 4mtD3mCerulean3+16 [16] and H2BD3mCerulean3+16 (Figure 5A,B). As shown in Figure 5B, 4mtD3mCerulean3+16 R% increased concomitantly to that of H2BD3mCerulean3+16, with different peak amplitude and kinetics. Considering the K_d_ of the two probes, the [Ca^2+^] peak values were about 1 μM in the nucleus and 25 μM in the mitochondrial matrix.

The same protocol was then applied to HeLa cells coexpressing the ER-targeted sensor D4ER [16] and the H2BD3mCerulean3+16 (Figure 5C,D). The R% calculated for D4ER decreased, while that of H2BD3mCerulean3+16 concomitantly increased upon histamine addition, indicating an effective ER Ca^2+^ release, which in turn resulted in fast elevation of cytosolic and thus nuclear [Ca^2+^] (Figure 5D).

## 3. Discussion

Ca^2+^ signaling modulates both physiological and pathological processes due to its spatiotemporal complexity, which is guaranteed by the coordinated action of a highly specialized and heterogeneous molecular Ca^2+^ toolkit and organelle Ca^2+^ dynamics present within the cell [1]. It is therefore obvious that the ability to simultaneously and quantitatively monitor changes in global and compartmentalized [Ca^2+^] are essential for in vitro and in vivo exploration of a variety of cellular processes. In an effort to extend the palette of available Ca^2+^ probes, we took advantage of a widely used FRET-based sensor characterized by a single K_d_, the Cameleon D3cpv [17], to generate a probe targeted to the nucleus with optimized properties. The bare addition of the nuclear targeting sequence H2B to D3cpv sequence, although retaining the advantages of ratiometric measurements, resulted in a sensor with low SNR of the donor at saturated [Ca^2+^] that compromises its usage in quantitative measurements. We therefore applied a strategy that we successfully exploited recently for similar probes [16], in which we demonstrated that the replacement of ECFP with mCerulean3 notably improved the brilliance and photostability of the cytosolic and mitochondria-targeted variants of D3cpv. However, as shown by us [16] and others [21], such donor replacement leads to a major reduction in the dynamic range. Although there is no clear explanation for this phenomenon, a recovery in the dynamic range has been obtained by elongating the glycine linker between CaM and M13 by 16 extra amino acids, thus increasing the flexibility of the probe structure [16]. As expected, donor replacement and addition of a glycine linker between the two Ca^2+^-responsive elements led to an increase in brilliance and photostability of H2BD3mCerulean3+16 probe at the expense of a reduction in dynamic range compared to H2BD3cpv. In this case, the higher dynamic range of H2BD3cpv compared to H2BD3mCerulean3+16 was only apparent as it was largely due to an abrupt reduction of the donor fluorescence at high [Ca^2+^] that made it indistinguishable from the background. This issue prevented the proper calibration of H2BD3cpv and thus its application for quantitative Ca^2+^ measurements. On the contrary, the improved photostability and the increased brightness of the donor made H2BD3mCerulean3+16 the best choice for quantitative imaging experiments. We successfully used this probe to simultaneously record Ca^2+^ kinetics from the nucleus and other subcellular compartments, i.e., mitochondria and ER. Surprisingly, the new nuclear probes displayed a double affinity for Ca^2+^, whereas its cytosolic and mitochondrial variants, as well as the purified protein, had a single K_d_. Finally, we identified additional evidence that nuclear and cytosolic Ca^2+^ are in equilibrium, corroborating previously published data [7,22,23,24]. It should be stressed that a nuclear Ca^2+^ probe is necessary for monitoring directly and quantitatively what happens to the cation concentration in the nucleoplasm. In addition, given the rapid equilibration between [Ca^2+^] in the two compartments, nucleoplasm Ca^2+^ signals can be used as a tool to contemporarily and comparatively monitor the dynamics of [Ca^2+^] in organelles, such as ER or mitochondria, and in the cytosol in the same cell and with the same type of probes. Obviously, although the nuclear Ca^2+^ dynamics mirrors global cytosolic Ca^2+^ changes, nuclear Ca^2+^ probes cannot properly report very localized cytosolic Ca^2+^ changes, such as those occurring in synaptic terminals or other cytoplasmic microenvironments [25].

Altogether, these results show that H2BD3mCerulean3+16 probe is a suitable FRET-based GECI for the quantitative analysis of Ca^2+^ dynamics in the nucleus and can be used in combination with other GECIs targeted to different subcellular compartments.

## 4. Materials and Methods

### 4.1. Constructs Generation

H2BD3cpv was generated as described in [13]. mCerulean3 was kindly gifted by M.A. Rizzo (Department of Physiology, University of Maryland School of Medicine, Baltimore, MD, USA). The H2BD3mCerluean3+16 construct was generated starting from 4mtD3mCerulean3+16 [16] and H2BD3cpv cDNAs. The 4mt sequence in 4mtD3mCerulean3+16-pcDNA3 vector was substituted by inserting H2B cDNA between the *Kpn*I and *Bam*HI restriction sites. H2B was amplified by PCR to add the restriction sites using the following primers:

F-5′-TCAAGCTT*GCCACC*ATGCCAGAGCCAGCG-3′

R-5′-CAAGTACACCAGCGCTAAGTGCGGCCGCGGATCCCG-3′

### 4.2. Cell Culture and Transfection

HeLa cells were grown in Dulbecco’s modified Eagles medium (DMEM) containing 10% fetal bovine serum, supplemented with l-glutamine (2 mM), penicillin (100 U/mL), and streptomycin (100 µg/mL), in a humidified atmosphere containing 5% CO_2_. Cells were seeded onto 18 mm diameter glass coverslips, and transfection was performed at 60–70% confluence using TransIT^®^-LT1 transfection reagent (Mirus Bio LLC, Madison, WI, USA) with 1 µg of cDNA. Fluorescence and confocal imaging were usually performed 24 h after transfection.

### 4.3. Confocal Analysis of mCerulean3 Fluorescence and Nuclear Localization

For TO-PRO-3 staining, after fixation with 4% paraformaldehyde (PFA) for 10 min, cells were permeabilized with 0.1% Triton X-100 in PBS for 3 min and washed and incubated with the dye (1 μM, dissolved in PBS) for 15 min. After PBS washing, coverslips were mounted using Mowiol (Sigma-Aldrich Saint Louis, MO, USA). Images were collected at a Leica TCS SP5 II confocal system equipped with a PlanApo 100×, numerical aperture (NA) 1.4 objective. For all images, pinhole was set to 1 Airy unit. Cameleon donor excitation was performed with an argon laser line (458 nm), and a white laser at 512 or 643 nm was used to excite the Cameleon acceptor or TO-PRO-3 dye, respectively. Confocal microscopy imaging was performed at 1024 × 1024 pixels per image with a 200 Hz acquisition rate. ImageJ program (Rasband, W.S., USA. National Institutes of Health, Bethesda, MD, USA) was used for image analysis. To compare the absolute fluorescence of ECFP and mCerulean3, the mean fluorescence intensity measured within a ROI was normalized to the cpV mean fluorescence recorded in the same ROI, maintaining the recording parameters for each channel constant in subsequent acquisitions. The presence of cpV in both probes allowed the exploitation of its fluorescence as a parameter to normalize for the protein expression level in each cell analyzed.

### 4.4. Fluorescence Microscope Settings for FRET Experiments

Live cells expressing the fluorescent probes were analyzed using a DM6000 inverted microscope (Leica Microsystems, Wetzlar, Germany) with a 40× oil objective (HCX Plan Apo, NA 1.25). LED excitation light (#LZ1-00UA00, LED Engin) was properly filtered at 410 nm through a band-pass filter, and the emitted light was collected through a beam splitter (OES S.r.l., Padua, Italy; emission filters HQ 480/40M (for ECFP) and HQ 535/30M (for cpV); dichroic mirror 515 DCXR). All filters and dichroic optics were from Chroma Technologies (Bellow Falls, VT, USA). Images were acquired using an IM 1.4C cool camera (Jenoptik Optical Systems) attached to a 12-bit frame grabber. Synchronization of the excitation source and the camera was ensured through a control unit run by a custom-made software package, Roboscope (developed by Catalin Dacian Ciubotaru at VIMM, Padua, Italy). Exposure time varied from 50 to 200 ms depending on the intensity of the fluorescent signal of the cells analyzed. Images were usually acquired every second, with the only exception being calibration experiments, in which one image was acquired every 5 s. During the experiment, the coverslip was placed into an open-topped chamber and cells were maintained in the proper medium. The extracellular medium (EC) was modified Krebs–Ringer buffer (mKRB), which contained (in mM) 135 NaCl, 5 KCl, 1 MgCl_2_, 0.4 KH_2_PO_4_, 1 MgSO_4_, 20 HEPES, 11 glucose, with pH 7.4 at 37 °C. The intracellular medium (IC) contained (in mM) 130 KCl, 10 NaCl, 1 MgCl_2_, and 20 HEPES, with pH 7.0 at 37 °C. All media were perfused through a temperature controller (TC-324B, Warner Instruments, Hamden, CT, USA) to maintain a temperature of 37 °C in the chamber. Preincubations were also performed at 37 °C.

Functionality experiments:

(1) ER-Ca^2+^ release protocol: Classical experiments started in EC containing 1 mM CaCl_2_; after perfusion with 600 µM EGTA, cells were stimulated by perfusing with 100 µM histamine and 20 µM CPA, when indicated.
(2) SOCE-activating protocol: Classical experiments started in EC containing 1 mM CaCl_2_; after 7 min of perfusion with EC containing 600 µM EGTA in the presence of 100 µM histamine and 20 µM CPA to empty the stores and maximize STIM1 oligomerization and translocation, cells were perfused with EC containing 2 mM CaCl_2_.

Dynamic range: In HeLa cells expressing the nuclear probes, R_min_ and R_max_ values were obtained upon permeabilization of the cells in Ca^2+^-free IC medium containing 600 µM EGTA and 100 µM digitonin for 60 s. The R_min_ was achieved by perfusing permeabilized cells with IC medium containing 600 µM EGTA, while the R_max_ was achieved with IC medium containing 3 mM CaCl_2_. Alternatively, in intact cells, R_min_ was obtained by incubation for 30 min at 37 °C in EC in the presence of 600 µM EGTA and 5 µM BAPTA-AM, while R_max_ was obtained by adding 5 µM ionomycin in EC containing 3 mM CaCl_2_. The dynamic range was calculated by dividing R_max_ (cpV/ECFP or cpV/mCerulean3 fluorescence intensity at saturated [Ca^2+^]) by R_min_ (cpV/ECFP or cpV/mCerulean3 fluorescence intensity at minimal [Ca^2+^]).

Photostability experiments: HeLa cells expressing each of the nuclear probes were illuminated every second using the same LED intensity at 410 nm. Images were acquired for 20 min at 1 Hz in two different FRET conditions: minimal FRET, which was obtained by bathing cells prepermeabilized with 100 µM digitonin in IC Ca^2+^-free medium containing 600 µM EGTA, and basal FRET, which was obtained by incubating intact cells in EC in the presence of physiological 1 mM CaCl_2_.

In situ calibration: HeLa cells expressing H2BD3mCerulean3+16 were permeabilized with 100 µM digitonin in IC Ca^2+^-free medium containing 600 µM EGTA for 60 s and then incubated in the same medium without digitonin. Cells were then perfused with an IC at pH 7 containing known Ca^2+^ concentrations. At the end of each experiment, a saturating CaCl_2_ concentration (3 mM) was applied. IC supplied with 500 µM BAPTA free acid (Thermo Fisher Scientific, Waltham, MA, USA) was used to achieve [Ca^2+^] < 0.5 μM for K_d_ evaluation. The free [Ca^2+^] was estimated using MaxChelator2.5 and checked by fluorimetric measurements with Fura-2. The data obtained were plotted as log_10_[Ca^2+^] (x-axis) and R% (y-axis) and fitted using Origin 8 SR5 (OriginLab Corporation). Fitting was performed using the following equation:

y = (Rmax1 × x^n1^)/(kd1^n1^ + x^n1^)+(Rmax2 × x^n2^)/(kd2^n2^ + x^n2^).

### 4.5. Buffer Titration with Spectrofluorometer

To evaluate the [Ca^2+^] of the IC medium used for the calibration experiments, 0.5 µM of Fura-2 (Fura-2 pentapotassium salt, Molecular Probes) was added to the solution and fluorescence was evaluated using the Perkin Elmer LS50B spectrofluorometer equipped with magnetic stirring (340 and 380 nm excitation intensity, 510 nm emission) at 37 °C. The [Ca^2+^] concentration was calculated as follows: [Ca^2+^]_free_ = 224 × Q [(R − R_min_)/(R_max_ − R)], where 224 nm is the Fura-2 K_d_, R represents the fluorescence intensity ratio F_λ1_/F_λ2_, in which λ_1_ is ~340 nm and λ_2_ is ~380 nm. R corresponding to the titration end points is denoted by the subscripts indicating the minimum and maximum [Ca^2+^]. Q is the ratio of F_min_ to F_max_ at λ_2_ (~380 nm).

### 4.6. Materials

Restriction and modification enzymes were purchased from Thermo Fisher Scientific (Waltham, MA, USA). Digitonin, histamine, CPA, DMEM, trypsin, L-glutamine, and penicillin were purchased from Sigma-Aldrich (Saint Louis, MO, USA). BAPTA and Fura-2 were purchased from Invitrogen, Thermo Fisher Scientific (Carlsbad, CA, USA). All other materials were analytical or of the highest available grade.

### 4.7. Data Analysis and Statistics

Off-line analysis of FRET experiments was performed with ImageJ software (National Institutes of Health). cpV and ECFP/mCerulean3 images were subtracted of background signals and distinctly analyzed after selecting proper ROIs on each cell; subsequently, a ratio between cpV and ECFP (or mCerulean3) emissions was calculated (R = F_530_/F_430_).

Data are presented as follows:

- A normalized R/R_0_, where R_0_ is the R value at the beginning of the experiment (t_0_) and R is the R value at each time (t) of the experiment;
- R% is calculated as R% = (R − R_min_)/(R_max_ − R_min_) × 100;
- [Ca^2+^] is calculated as described in [19].

All data are representative of at least three different experiments. Unless otherwise stated, numerical values presented throughout the text refer to mean ± SEM (*n* = number of independent experiments or cells). Statistical significance (*p* < 0.05) was determined by Student’s *t*-test (two-sided) for comparison of two groups with normal distribution and Wilcoxon test for comparison between two groups with non-normal distribution.

## Figures and Tables

**Table 1:** In situ properties of H2BD3mCerulean3+16. DR, dynamic range; K_d_, dissociation constant; N, Hill constant. Data are presented as mean ± SEM of *n* ≥ 7 cells.

H2BD3mCerulean3+16	DR	K_d_ (µM)	N
K_d1_	2.95 ± 0.06	0.03 ± 0.01	4.1 ± 4.6
K_d2_		7.57 ± 0.81	0.8 ± 0.06

## Data Availability

Data are contained within the article.
